# Immunohistochemical searching for estrogen and progesterone receptors in women vocal fold epithelia

**DOI:** 10.1016/S1808-8694(15)30593-0

**Published:** 2015-10-18

**Authors:** Oswaldo Angel Bellido Rios, Andre de Campos Duprat, Adriana Ribeiro dos Santos

**Affiliations:** 1 M.S. Student. Instructor at Santa Casa de São Paulo, Otorhinolaryngologist.; 2 PhD. Professor. Head of the Otorhinolaryngology Department - School of Medical Sciences - Santa Casa de São Paulo.; 3 Gynecologist and obstetrician. Second Assistant - Department of gynecology and obstetrics - Guarulhos General Hospital -Santa Casa de São Paulo. School of Medical Sciences - Santa Casa de São Paulo.

**Keywords:** estrogen, hormones, larynx, progesterone

## Abstract

Larynx is extremely sensitive to endocrinologic changes. Most vocal fold mucosa alterations are caused by changes in vocal fold liquid content and its epithelial changes. Estrogen and progesterone interfere and change this liquid content in the vocal folds. Our goal with the present paper is to study the presence of estrogen and progesterone receptors on vocal fold epithelium in 19 vocal fold epithelium specimens that did not present any indication of disease, especially inflammatory disease. We discarded those cases of patients above 40 years of age and those below 15.

**Results:**

we found progesterone receptors in 18 of the 19 patients. The progesterone receptors are located both in the nucleus and the cytoplasm of cells, and mainly in the basal layer. There was no report of estrogen receptors present in the vocal folds.

**Conclusion:**

Vocal fold epithelium bears progesterone receptors, in the cytoplasm and in the nucleus. We did not find estrogen receptors in the epithelia of the vocal folds investigated.

## INTRODUCTION

Hormones are molecules secreted onto the blood current that usually have their biological effect in sites away from where they were produced. They cause growth, differentiation and functionality in different target organs. Hormones effect on target organs is due to their bindings with intracellular receptors, modulating gene expression and, consequently, the synthesis of specific proteins^1^.

Hormonal receptors are being currently investigated in almost all anatomical regions, such as the breast, bones, testicles, brain, lacrimal glands, heart, ovaries, endometrium, among others^2^. Despite all of this, the interaction between the endocrine system and the larynx is surprisingly not studied enough, even knowing of the great susceptibility the larynx has towards hormonal effects^3^.

Such interaction is proven by clinical and empirical events that occur in the larynx, such as voice change, pre-menstrual syndrome and vocal alterations during menopause.

The effects of sex hormones is clearly seen in voice change, when there is a differentiation between male and female voices, and when a kid's voice turns into an adult's voice^4^. Menstruation may prevent people who have their voice as a working tool to sing, because in the days immediately before menstruation and ovulation, hormonal alterations interfere in their voices^5^. During this period, female singers are encouraged by conductors and singing tutors not to perform, because of a drop in voice quality^6^.

Based on these clinical observations, some researchers such as Abitbol et al.^7^ and Caruso et al.^8^ used cytology studies to assess the alterations hormones would cause to vocal cords. These authors noticed that cell alterations that happened in the uterus cervix during the menstrual cycle were the same ones that happened on the vocal cord mucosa, following the different hormonal actions of estrogen and progesterone. They showed that in fact, vocal fold epithelium may suffer the action of sex hormones. Abitbol et al.^7^ did their study before, during and after the menstrual cycle. Caruso et al.^8^ studied female patients who were in menopause and had atrophy of their uterine cervix and vocal cords before and after hormonal replacement therapy. These cell studies on the action of sex hormones in the larynx were the necessary basis for the next studies about hormonal receptors in vocal cords.

There are only two papers that approach hormone presence in vocal cords: Piatkowski et al.^9^ used immunohistochemistry to study the presence of estrogen and progesterone receptors in the larynx of patients with cancer and in normal individuals. Hormone receptors were not found in the vocal cords, but rather in the supraglottic region. Newman et al.^10^ also studied estrogen, progesterone and testosterone receptors in vocal cords. The study was carried out in cadavers of both genders. Progesterone and testosterone receptors were positive in all the cases. Only 5 of 18 patients were positive for estrogen. These two papers showed that there is little estrogen receptor expression in the vocal cords, contrary to what was expected since histology studies proved large hormonal action on vocal cords, especially that of estrogen.

The possible ambiguity among cytology studies of estrogen and progesterone action on the larynx and the little expression of hormonal receptors on vocal cords led to the receptor's research carried out in the present paper. The goals have been to asses the presence of estrogen and progesterone receptors on the epithelium of women's vocal folds and differentiate their intracellular location.

## MATERIALS AND METHODS

This paper has been submitted to and approved by the Ethics Committee of the School of Medical Sciences of the Santa Casa de São Paulo, protocol # 022/05.

We studied slides from patients followed at the Department of Otorhinolaryngology of the School of Medical Sciences of the Santa Casa de São Paulo. Theses slides were obtained retrospectively after reviewing the charts of patients who had previously undergone vocal cord surgery from January through December of 1999.

The slides chosen presented normal epithelium, and those that had some acute or chronic inflammatory process were discarded.

We also excluded from the study, the slides from patients above 40 years of age and below 15 years old.

The slides selected bore the following problems: mucous bridge (one case), submucosal cyst (13 cases), hemangioma (one case), bridle (two cases) and larynx biopsy (two cases).

The slides selected were histologically evaluated embedded in paraffin and fixed in formalin. The specimens were then sectioned at a thickness of 5 to 6mm. The cross sections were then hydrated, had the paraffin removed were and embedded in a 0.01 mols/L saline buffer in pH of 7.4 and treated with 0.1% trypsin for 30 minutes at 37ºC. The specimens were covered with hydrogen peroxide for 5 minutes and after that were incubated separately with primary estrogen and progesterone antibodies, during one night, at 4ºC. The reaction was developed with diaminobezidine at room temperature for 10 minutes and, after that, the procedure was repeated for the secondary antibodies. Immunoreactiveness was studied under light microscopy.

The following primary antibodies were used: Estrogen Receptor Antibodies Lyophilized Monoclonal (NCL-ER-6f11) and the Progesterone Receptor Antibodies Lyophilized Monoclonal (NCL-PGR-312) from Novocastra. The secondary antibody used was the Strept complex. ABComplex/HRP, Rabbit/Mouse (code k0492) from Biogen (DAKO). The slides were dyed with hematoxylin eosin after the use of antibodies.

The slides were studied according to the positiveness of estrogen and progesterone receptors and their presence was verified as to its intracellular location (nucleus or cytoplasm). Data was statistically evaluated by means of the 95% confidence interval.

## RESULTS

We found 19 cases of normal vocal cord epithelium for the study of estrogen and progesterone receptors.

1-Age

Patients’ ages varied between 25 and 34 years. Mean age was of 31.58 years.

2- Estrogen

We did not find estrogen receptors on the vocal cords epithelium, both in the cytoplasm and in the cell nucleus. Lack of estrogen p= (0/19) =0; p<0.0000001.

3- Progesterone

Progesterone was positive in 18 of the 19 cases studied in the epithelium of the vocal folds. The presence of progesterone was of p = (18/19) = p=0.952; p<0.05. The only negative case came from a bridle on the vocal cords. The remaining cases: mucous bridge (one case), submucosal cyst (13 cases), hemangioma (one case), bridle (one case) and larynx biopsy (two cases) were progesterone positive.

**Figure 1:**
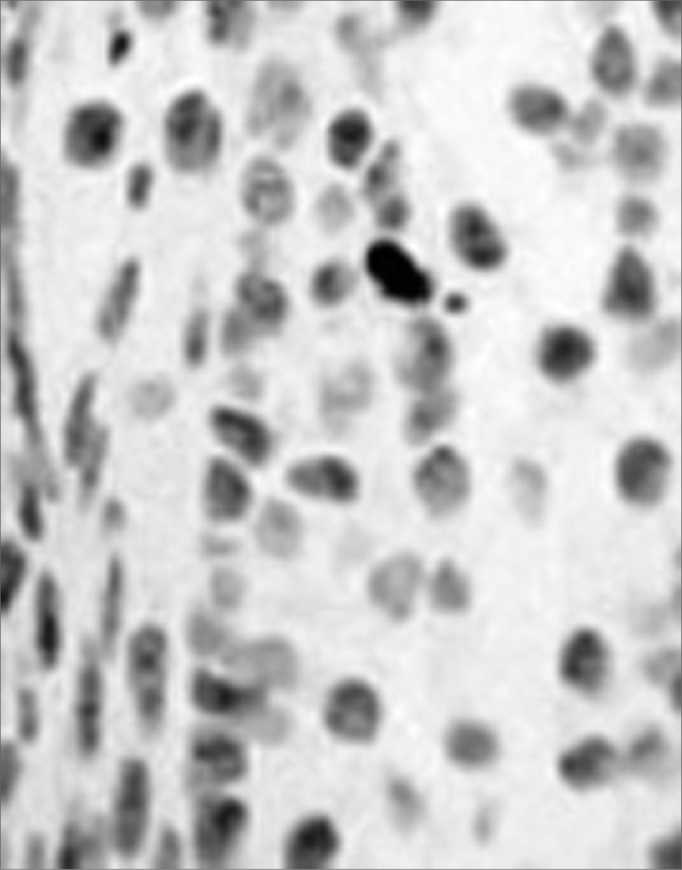
Vocal fold without immunohistochemistry - Figure 2. LM.HE 318x. Vocal fold cross-section immediately anterior to that of Figure 1, without the use of immunohistochemistry.

**Figure 2:**
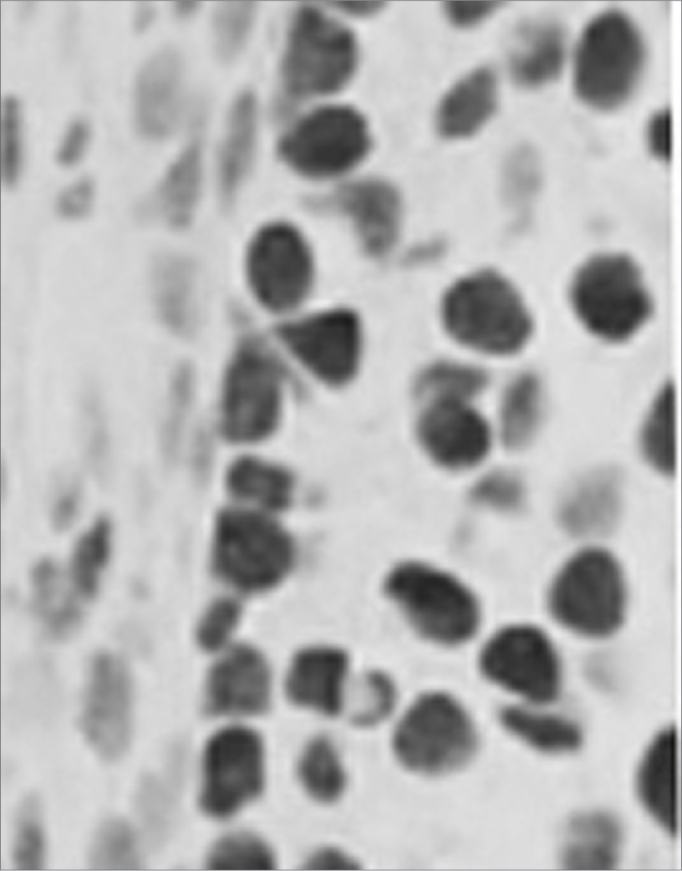
Progesterone positive - Figure 1. LM. HE. 318x. Vocal fold cross-section showing progesterone receptors. Positiveness is seen by means of this red-brownish dye.

**Figure 3:**
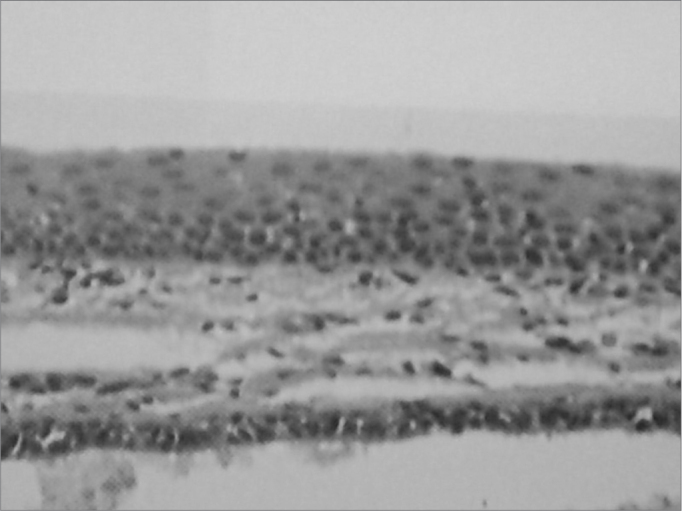
Negative for estrogen receptors - Figure 3. LM. (63x) HE. Normal vocal fold epithelium; negative for estrogen receptors.

**Figure 4:**
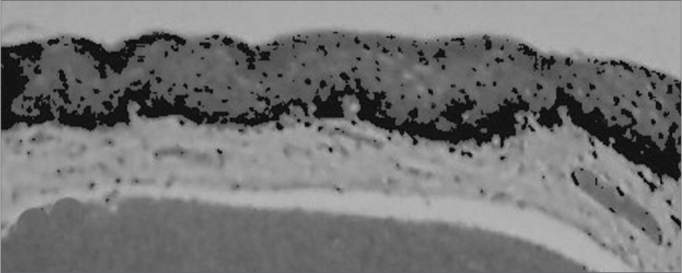
Positive for progesterone receptors - Figure 4. MO.HE.(63x) Normal vocal fold epithelium; positive for progesterone receptors. Notice the brownish color of the vocal fold epithelium.

a) Location in the epithelium: the 18 positive cases were studied as to their epithelial location: basal layer or intermediate zone. In order to do that, we counted the basal layer cells in a visual field that had the reagent and the cells which were not dyed. Of the 18 cases studied, we noticed that only 4.34% of the cells were not positive. In the intermediate layer, there was a less standardized pattern, negative in 3 cases, with one or two positive cells in 13 cases and in two cases of total positiveness, with only 2 cells in one slide that was not positive and 3 cells which were not positive in another slide (Pictures 1, 2, 3 and 4).

Picture 1) LM.HE. (318x) Positive for the progesterone receptor (notice the brownish color). 2) LM.HE. (318x) vocal fold, showing an immediately anterior cross-section, without dye in order to assess progesterone positiveness (bluish color).3) LM. (63x) HE. Normal vocal fold epithelium, negative for estrogen receptor. 4) MO.HE. (63x) Normal vocal fold epithelium, positive for progesterone receptor. Notice the brown color of the vocal fold epithelium.

b) Intracellular location (Nucleus x Cytoplasm)

All 18 positive cases had receptors in their cell nuclei. Cytoplasm was positive in 16 of the 18 cases.

## DISCUSSION

The results presented in this paper show that sex hormones do act in the vocal folds; however, in a different way. These characteristics may be important in understanding and treating different larynx disorders.

In the present investigation, the research was carried out by immunohistochemistry, which was used because it is a typical tumoral maker^11^, since it bears high sensitivity and allows morphological tissue evaluation. It is also a semi-quantitative test, bearing lower costs that the ligandin binding test, and it is easier in slide handling. It also marks cell development lineages and requires less fresh tissue to be carried out^11^. Until 1990's, estrogen receptors investigation was carried out by the ligandin binding text in fresh tissue. More recently, immunohistochemistry has become the method of choice to determine the presence of such receptors, since it is clearly superior to the ligandin test^12^.

As to age, we excluded menopause patients, and we considered only those patients above 40 years of age. At menopause there is less estrogen, progesterone and testosterone hormone expression^13^. Such behavior could influence results in this study group, and their exclusion was necessary for statistics sake.

Patients below 15 years of age were also excluded. Hormonal receptors’ behavior at this age range is markedly different. It is believed that the number of receptors in the pre-pubertal phase be higher when compared to any other age. Clinically, we observe in males that estrogen use in the fetal or neonatal period may cause reproductive disorders such as cryptorquism, epididymal defects, infertility, and increase in testicle cancer incidence^14^. In females, menarche is of genetic origin; it is influenced by sex hormones and mediated through estrogen receptors^15^. Such genomic action is more intense in the pre-pubertal period, and this fact could impact our results.

Piatkowski et al.^9^ and Newman et al.^10^ did not consider age in their studies. They included all patients, even those below 15 years and those after menopause. Piatkowski et al.^9^ also included all patients in their study group, as a matter of fact, all their patients were above 45 years of age. This may very well be one of the reasons as to why they did not find estrogen, progesterone or testosterone receptors in the vocal folds. The presence of estrogen receptors in Newman et al.'s investigation^10^ was positive only for those patients below 15 years of age, including patients in these two age ranges, when there is marked hormonal changes, may account for the different results and be a bias factor in these two investigations.

In our study, the disorder diagnosis did not impact results. Diagnosis: mucosal bridge, submucosal cyst, submucosal hemangioma and biopsy did not interfere in the results attained. The only case in which we did not have positiveness was for bridle, and the epithelium was normal.

In our investigation we did not find estrogen receptors. Such result was also negative for Piatkowski et al.^9^. Newman et al. found positiveness in only five of the 18 cases studied^10^. Alpha (Eα) and beta (Eβ)^16^ estrogen receptors were jointly investigated in our study, since we were not interested in their differentiation. The alpha estrogen receptor has a greater predisposition for the development of malignant tumors; and as for the beta (Eβ), there is a greater likelihood of the person developing pre-malignant lesions.

Results from Piatkowski et al.^9^ and Newman et al.^10^ are in disagreement in relation to a cytology paper published by Abitbol et al.^7^ and Caruso et al.^8^, who showed a relevant estrogen presence.

Caruso et al.^8^ studied menopause women who had not undergone hormonal replacement therapy. In their vocal fold epithelium there were signs of atrophy and dystrophy before treatment. After hormonal replacement, these signs disappeared. The use of medication caused epithelial atrophy normalization, both in the uterine cervix and in the larynx, thus proving the estrogen action in the vocal folds.

Abitbol et al.^7^ found epithelial changes in the vocal folds and in the uterine cervix in all phases of menstruation, proving that the larynx is an organ that responds to hormonal changes in each one of the menstrual cycle phases.

Based on these data, we may deduct that there is estrogen action in the larynx; however, such action may not be present in this epithelium as previously believed. These receptors may be located in other sites such as the vocal fold muscles. It is known that the inner portion of the thyroarytenoid muscle is the major site of androgenic hormonal action^17^, this could also be the action site for estrogen. Wu et al. noticed that estrogen receptors may be present in neuromuscular synapses present in larynx intrinsic muscles^18^.

Piatkowski et al.^9^ noticed that hormonal receptors are preferentially present in the supraglottic region. Such region bears a number of glands implicated in the production of laryngeal secretions. These hormone-rich secretions could soak the vocal folds and cause the alterations seen in these cytology studies.

Another factor to be considered is that progesterone receptors inhibit their estrogenic counterparts^19^. In our study, we saw positiveness for progesterone receptors in 18 of the 19 patients investigated. By means of self-regulation mechanisms, estrogen receptors have their presentation blocked. On the other hand, estrogen receptors may indeed be lacking in the vocal folds, as it is seen in other organs, such as the liver^20^.

As far as progesterone is concerned, there was positiveness in 18 of the 19 cases, representing their important presence in the vocal folds epithelium, in agreement with the results found by Newman et al.^10^, who found a relevant presence of progesterone receptors in the vocal folds. Contrary to that, Piatkowski et al.^9^ did not find progesterone receptors in the vocal folds. As it happened to the estrogen receptors, the aforementioned author did not find positiveness for progesterone in their patients above 54 years. They would include menopause patients, who have a lower progesterone receptor expression.

In almost all organs, progesterone receptors are the main structures responsible for commanding cell activity, including the endometrial glands, where estrogen and progesterone act in a synergic fashion^21^. This marked progesterone action is caused by its direct action on cell nuclei transcription, the main cell activity command center^22^.

In our investigation we studied progesterone presence in both the nucleus and the cytoplasm. The test was positive in the nuclei for all the cases; and it was only in 3 of the 18 cases that it was not positive in the cytoplasm. The goal in differentiating between nuclei and cytoplasm is that these two types of receptors have different behavior^22^. The receptors located in the nucleus are able to modulate the hormones, inhibiting or stimulating them, and thus, controlling their different functions in the different body tissues; therefore, controlling their different functions in the different body tissues; thus, commanding the different physiologic effects^23^. The so called non-nuclear cytoplasmic action is related to the quick vasodilatation and vascular response inhibition towards tissue damage^23^.

In our study we found progesterone receptor dominance in the epithelial basal layer. This layer is the one with the most proliferative activity. This proliferative activity was noticed by Van Cruchten^24^ in the epithelium of dogs and was related to progesterone activity. This progesterone-induced proliferative activity was present with an increase in mitotic activity, angiogenesis and crypts. Nogushi et al.^25^ noticed intense progesterone-induced extracellular matrix production activity from the basal cell layer. Since extracellular matrix production and epithelium proliferative activity are conditions present in both the normal and pathological larynx activity, this progesterone action in the basal layer has to be better investigated.

The clinical importance of our findings here is that progesterone can cause vasodilatation, hemorrhage and alterations in the epithelial surface lubrication^26^. Sex hormones are implicated in the aquaporin channel regulation^27^, which are a family of proteins from cell membrane channels that facilitate water transport^27^. Thus, progesterone is one of the most important factors in regulating epithelial surface lubrication, since water output from the epithelium is directly related to its presence. It has been proven that in patients with dysphonia caused by stress and tiredness of the vocal folds, there is symptoms’ worsening because of an increase in mucous viscosity; increase in mucous aggregation and also by the roughness on vocal fold surface^28^. Larynx mucous viscosity influences vocal fold vibration^28^. Nakagawa et al.^29^ applied fluids of different viscosities on the vocal folds of dogs and noticed that the higher the viscosity, the less is vocal fold range of vibration and smaller glottic contact area, thus affecting vocal fold surface movement. Knowledge about aquaporins is already being used for the treatment cerebral edema with progesterone (Guo et al.)^30^.

By the results found in the present investigation, we believe the larynx is a target for progesterone action, as far as vascular action is concerned. During the menstrual cycle, new capillaries are created from the already existing microvasculature. These new capillaries suffer maturation and remodeling, forming a new vascular network at each cycle. (Albrecht and Pepe)^31^. Kayisle et al.^32^ stated that blood vessel growth and regression are regulated by sex hormones through the menstrual cycle and the major agent responsible for this angiogenic proliferation is progesterone and estradiol. Another investigation found unequivocally the presence of progesterone receptors in the vessels of many different tissues, including the placenta, where progesterone is the major factor involved in angiogenesis (Cudeville et al.)^33^.

The knowledge acquired by the present investigation regarding sex hormonal action on the larynx is essential in understanding the different larynx disorders. Hormones affect the larynx in different ways, especially sex steroids, and must be always assessed when one has a patient with vocal complaints, and should be part of the therapeutic arsenal of all otorhinolaryngologists.

## CONCLUSION

Progesterone receptors are present in the basal layer of the vocal fold epithelium and are located in cell nucleus and cytoplasm. Estrogen receptors were not present in vocal fold epithelium.
